# “You Can Change the World With a Haircut”: Evaluating the Feasibility of a Barber-led Intervention for Men of Black and Ethnic Minority Heritage to Manage High Blood Pressure

**DOI:** 10.1177/21501319231168336

**Published:** 2023-05-06

**Authors:** Nicola Thomas, Catriona Ewart, Debi Lewinson Roberts, Andrew Brown

**Affiliations:** 1 London South Bank University, London, UK; 2 Croydon BME Forum, Croydon, UK

**Keywords:** underserved communities, community health, prevention, blood pressure, barbershops

## Abstract

**Background:**

People of Black, Asian and minority ethnic (BAME) heritage have a higher-than-average incidence of, and mortality from hypertension and stroke. Therefore, it is important to identify new settings for engaging people at risk of high blood pressure (BP).

**Aim:**

This feasibility study aimed to evaluate if barbers in a London borough can support and educate men of BAME heritage to manage their BP. Following UK Medical Research Council guidance, the RE-AIM (reach, effectiveness, adoption, implementation, maintenance) framework was used to guide study objectives and feasibility outcomes.

**Methods:**

We collaborated with 8 barbers who were part of an existing BAME barber network. Barbers were trained online (1.5 h) and face-to-face (3 h) to provide BP healthcare advice and take customers BP readings. Qualitative field notes were collected to assess how best to recruit and train barbers, and to understand how to maintain motivation and retention of barbers. BP readings were recorded between June 2021 and March 2022.

**Results:**

Both online and face-to-face training were effective, however, greater focus on how to start conversations about BP with clients was needed. We found that motivation, incentivization and regular contact with barbers were important for recruitment, retention, and sustained BP measurement. Obtaining BP readings was challenging due to client concerns about recording their data and the impracticalities of recording results. We captured 236 BP recordings, of which 39 (16.53%) were over 140/90 mmHg; of these, 5 were over 180/100 mmHg.

**Conclusion:**

The combined data showed that educating barbers to take BP readings and deliver healthcare advice about BP is a viable intervention for rollout in a large-scale study. It has demonstrated the need to identify strategies to motivate barbers for sustained recruitment and retention, as well as further efforts to build trust among customers for long-term BP surveillance.

## Introduction

Hypertension is a major public health concern due to its worldwide high prevalence and increased risk of cardiovascular disease and kidney disease.^1^ However, people of Black, Asian, and Minority Ethnic (BAME) heritage in the UK are disproportionately affected by incidence of and mortality from hypertension and stroke.^2^ The reasons for this are multi-factorial. However, a lack of awareness and poorer engagement with traditional healthcare settings are potential contributory factors.^3,4^ Evidence further suggests that African-Caribbean men have less frequent contact with the health care system compared with black women and thus lower rates of hypertension detection, treatment, and control.^5^ One potential explanation for the lack of Black men receiving treatment for hypertension is the overall distrust for health care systems and research.^6^ It is therefore important to identify new settings for engaging people at risk of high blood-pressure who do not frequent usual health care.

Health promotion in partnership with barbershops has proven to be an effective strategy to reach populations most at risk of health disparities and build trust among minority communities.^7^ Barbershops have been successful venues for health professionals to deliver health promotion and disease prevention for prostate cancer, HIV testing for Black men, and sexual health education among BAME communities.^8-12^ Recently, Victor et al,^13^ conducted a randomized controlled trial enrolling black male clients from black-owned barbershops assigned to a pharmacist-led intervention for blood pressure control and management. The barber-shop intervention found that at 6 months, mean BP fell by 27.0 mmHg in the barber plus pharmacist group and by 9.3 mmHg in the barber-only group. The mean reduction was 21.6 mmHg greater with the addition of the on-site pharmacist. A BP of less than 130/80 mmHg was achieved among 63.6% in the barber plus pharmacist group versus 11.7% in the barber-only group (*P* < .001). However, previous research indicated that higher and more sustained customer participation in barbershop BP monitoring can be achieved when delivered by barbers compared to research personnel.^14^ Therefore, it is the trusting relationship that barbers have with their clients, that lends itself to an intervention delivered by barbers.

## Aims and Objectives

Barbershops have been effective venues to promote sexual health and HIV prevention among ethnic minority groups, and also for promotion of blood pressure awareness and control by community nurses in barbershops.^15^ However, to our knowledge, no studies have evaluated the feasibility of delivering a barber-led intervention for hypertension among BAME men in the UK. Historically, barbershops in African American communities have been an important cultural institution^16^ and are therefore a culturally and contextually appropriate establishment to reach men of BAME heritage, providing a novel setting to address health disparities. Therefore, the aim of this study was to assess the feasibility of a barber-lead intervention to support and educate men of BAME heritage to manage their blood pressure in Croydon, London.

## Methods

As recommended by the Medical Research Council,^17^ the RE-AIM framework^18^ was used to determine the feasibility of implementing the Barber Blood Pressure Project, a barber-led health intervention for BAME men at risk of high blood pressure. The project was a multi-site, single-cohort feasibility project conducted between January 2021 and March 2022.

We engaged with barbers to discuss the study set-up and intervention delivery supported by members of the Croydon BME Forum (https://cbmeforum.org/) and Off the Record (https://www.talkofftherecord.org/). This support included co-production of intervention, identification of barbershop sites, and liaison with barbers. Our project team comprised one project manager (7 h/week), one individual for barber liaison (3 h/week), the CEO from the Croydon BME Forum and an academic kidney nurse. The intervention was complementary of the existing community healthcare program offered by the BME Forum (https://cbmeforum.org/the-wellness-centre/).

### Recruitment

Barbers (n = 8) from an existing BAME mental health barbers’ network in south London, UK, were approached and conveniently recruited from 5 local barbershops, selected on location and positive response to invitation. In 2017, Public Health England^19^ estimated hypertension in adults over 18 years in Croydon to be 23.8%. The borough has a diverse population comprising of 18 ethnic categories, with 8.6% identifying themselves as African-Caribbean and 6.8% as Asian.

### Intervention Procedures

Due to the impact of COVID-19, the project was delayed, and barber training commenced in February 2021 with the hope that barbershops would reopen in Spring 2021. Following UK government restriction guidance, barbershops reopened in April 2021.

Training Part 1 took place in February 2021 online via Zoom and consisted of: the background to the project, the barber’s potential involvement, and discussion about the barbers’ input into the intervention and anticipated consultation time per customer. There needed to be an opportunity for barbers to have a realistic overview of their expected commitment to the project, an opportunity to ask questions and understand the benefits for their clientele. Training Part 2 was scheduled for April 2021 and was conducted in person. The barbers were taught how to approach clients to participate and offer BP checks with each haircut to eligible customers: males over 18 years of BAME heritage. Barbers were taught how to measure and interpret BP, record BP readings, and encourage clients to seek further healthcare advice where appropriate.

Barbers were advised to put the A2 BP poster in their shop window to attract footfall and promote the project within their communities. Barbers were provided with client cards to distribute BP advice to customers, with space to note 4 consecutive BP readings. Barbers were also provided with notebooks to record clients BP readings. It was difficult to define a target number of BP readings, but it was initially assumed (pre-pandemic) that each barber would have between 10 and 50 clients per day, dependent on the number of barbers in each barbershop.

The barbers were contacted fortnightly via telephone and WhatsApp to remind barbers to forward any BP data collected from clients. Where possible, barbershops were visited in-person fortnightly. Incentives for barbers during the project included training programs, networking opportunities, awareness of grants they could apply for, and social media training.

### Data Collection

Qualitative data were obtained from observed interactions as field notes throughout the project. Field notes were collated from liaison with barbers and stakeholder organizations, informal discussions with barbers, discussions with stakeholder organizations, interactions via WhatsApp, telephone and email, and when relevant observable actions occurred. Field notes, although underutilized in research and innovation projects,^20^ which provide a rich understanding of the context of intervention outcomes.^21^ Informal discussions were conducted with barbers at fortnightly site visits.

Preliminary quantitative data were collected to assess the number of blood pressure readings recorded by barbers and the blood pressure values of clients visiting barbershops. Barbers maintained a hand-written record of client BP readings and shared data with the project team fortnightly via WhatsApp images.

### Feasibility Outcomes

The RE-AIM dimensions were assessed independently using data qualitative and quantitative data collection. Table 1 provides an overview of the variables assessed under each RE-AIM component and the data source used to assess each variable.

**Table 1.** RE-AIM Evaluation Measures and Data Sources.

Evaluation level	Measures	Data Source
Reach	The number of barbershop sites and barbers recruited to deliver the intervention	Informal discussions and field notes
	The number of client BP readings recorded by barbers	Barber data returns
Effectiveness (potential and perceived)	Barbers’ views and feedback on the training provided	Informal discussions and field notes
	Barbers’ views and feedback on the project’s effectiveness	Informal discussions and field notes
Acceptability	Barriers and facilitators to barbers participating in the project	Informal discussions and field notes
	Barriers and facilitators to clients participating in the project	Informal discussions and field notes
Implementation	Extent to which the project objectives were met	Barber data returns
	Degree to which the project activities were administers	Informal discussions and field notes; observations
	Number and types of adaptions made to the project	Informal discussions and field notes
	Barriers and facilitators to implementation	Informal discussions and field notes
	Time and financial costs of the project	Project team
Maintenance	Reasons for the lack of implementation	Informal discussions and field notes; observations
	Resources necessary to guide monitoring and adaption of the project long term	Project team

The intervention was reviewed using qualitative data from field notes, site visits, and general observations.

## Findings

### Reach

In total, 8 barbers were recruited for the feasibility project. Five male barbers were recruited from the existing BAME Barbers Network WhatsApp group set up for a previous mental health project. Barbers were of African and Caribbean (n = 6), Greek Cypriot (n = 1) and Mauritian (n = 1) heritage and all had a large BAME clientele group.

By the project end date (March 2022), barbers had successfully recorded 236 BP readings. Of the available recordings, 39 (16.53%) were over 140/90 mmHg; of these, 5 were over 180/100 mmHg indicating very high blood pressure. As no demographic data were collected from clients attending barbershops, it was not possible to ascertain the populations reached by the intervention.

### Effectiveness

Overall, training was found to be effective both online and face-to-face. Barbers particularly enjoyed learning about the practical elements of BP monitoring: using the BP machines and interpreting BP readings. However, barbers reported that there needed to be more focus on how to initiate conversations with their clients about the project and BP health.

Throughout the project, communication via WhatsApp and engagement with the project decreased. The project manager found that the level of engagement was higher with barbers when attending sites in-person and interacting on a one-to-one basis. Although no barbers outrightly withdrew from the project, only 2 barbers sustained consistent BP monitoring throughout the project. Four barbers did become demotivated and 2 stated they had more pressing priorities. As no follow-up data were recorded on the client cards, it was not possible to ascertain whether the project impacted the number of clients attending the doctors following BP monitoring and advice at barbershops. This is planned for a future project.

### Adoption

Initially, barbers were optimistic about participating in the project. Intervention adoption was potentially limited due to the post COVID-19 impact on customer numbers and reduced footfall, which resulted in fewer opportunities for BP monitoring. As a result, we provided barbers with incentives to boost motivation by informing barbers of available training, networking, and business development opportunities. The most effective training incentive was a social media and Instagram training workshop which 4 barbers attended.

Due to the ongoing COVID-19 disruptions, project adoption among barbers was challenging. Barbers reported feeling anxious about the security of their business and whether clients would return as customers following lockdown. We found that the motivation of barbers was a key factor in barbers adopting the intervention. During the training, one barber was particularly enthusiastic and said “*you can change the world with a haircut.”* This same barber continued to demonstrate high levels of motivation throughout the project and returned the highest number of BP readings compared to the other barbers. One barber participated in a live Sky News interview, a success and a morale boost for all barbers to see their efforts on national television.

### Implementation

There were several barriers to implementing the project. Although posters were created, not all barbers used them to promote the project as intended. While client cards were provided and designed for consecutive BP readings at each visit to the barbershop, barbers reported keeping the cards to note BP readings and giving their clients verbal advice instead.

COVID-19 affected barbers’ motivation to implement the project as they were concerned about their business, and some were also forced to seek work outside the sector. Feedback from barbers revealed concerns about annoying their customers by repeatedly asking them to participate in the BP testing. Barbers further reported that some clients were resistant to having their BP tested, with clients saying they did not feel ill and therefore felt they did not need their BP monitored. Barriers to recording all BP readings taken were partly due to clients’ wariness in recording their data and the practicalities of writing or recording results.

Overall, the project was implemented as planned, with some minor changes. In Autumn 2021, the project lost momentum as barbers were felt demotivated. Therefore, additional funding was sought from a medical technology company to provide technical equipment to facilitate BP monitoring, and to provide additional incentives for barbers. Funding was approved in December 2021 and incentives (£5 per BP reading) were provided to the 2 barbers who initially demonstrated the highest level of engagement, then opened to the other barbers. The additional incentives proved effective and resulted in 189 (80%) of the BP readings recorded by the barbers during the final 3 months of the project.

Regarding cost in time to barbers, it was clear that barbers were focused on their business. While the training sessions outlined to barbers that BP monitoring would take 5 min per customer and could be completed alongside a haircut, barbers felt they did not have time to engage in the project or monitor BP readings. Additionally, a substantial amount of time was spent conducting fortnightly site visits and regularly needing to follow-up with barbers for BP readings. Modest funding was provided to cover staff wages (project manager 6 h/week and barber liaison role 3 h/week), travel costs for site visits, meeting venue costs, and project resources. The intervention implementation cost was relatively low: BP machines were £30 per machine and backfill time for barber’s 1.5-h training was £150 per barber attending both training days.

### Maintenance

None of the implementation sites have formally continued to take BP readings at the end of the project, due to lack of funding. However, further funding applications to extend the project across other areas of the UK have been submitted. The aim is to revise the barber recruitment process and training program (5 barbers in each location), understand the barriers and drivers to motivation further, ensure we reach blood pressure reading targets and track outcomes/experiences of those recorded as having high/very high blood pressure.

## Discussion

This study used the RE-AIM framework to evaluate the feasibility of delivering a barber-led health intervention to support and educate men of BAME heritage to manage their blood pressure. We recruited 8 barbers who successfully recorded 298 BP readings from BAME male customers across 9 months. Several contextual and individual barriers to BP monitoring in barbershops, including COVID-19 disruptions and customer concerns about their data being recorded. Although engagement was sometimes inconsistent, we found that motivation was key to the intervention’s success. Our most successful barbers had some connection to BP management, either from lived experience or previous job roles.

Barbers faced unprecedented barriers associated with ongoing COVID-19 disruptions hindering the intervention implementation. While barbers were initially enthusiastic about the project and the ability to positively impact other men of BAME heritage, a perspective consistent with previous barbershop studies^12,22^ barbers soon became demotivated. As such overall reach of the study was relatively low. However, consistent with previous barbershop studies, where incentivization resulted in positive outcomes,^14,23^ the supplementary funding increased barber participation and BP monitoring.

Contradictory to previous studies suggesting that barbers are well-positioned in their community to facilitate trust around health promotion,^7^ customers remained cautious about having their data recorded. Additionally, feedback on intervention training indicated that more focus on facilitating conversation on BP monitoring was needed, which could also incorporate strategies to establish trust between barbers and customers, as previously demonstrated.^10^ Luque et al^24^ provided a culturally sensitive “tool kit” to barbers delivering prostate cancer education, including brochures, posters, a DVD, talking points, and a community resource guide.

## Limitations

This was a feasibility study with limited resources and, thus, several limitations. We only enrolled 8 barbers from one geographic location using convenience sampling from an existing BAME network established for a previous mental health intervention, which did not translate into high engagement in the BP project. Consistent with feasibility studies, there was no control group included. However, previous research indicated higher and more sustained customer participation in BP monitoring was achieved when delivered by customers’ barbers compared to research personnel.^14^ Findings indicate that individual and contextual differences influence barber engagement and customers’ willingness to participate. No demographic or follow-up data were collected from customers, thus limiting the evaluation of the intervention reach.

## Future of the Barbershop Blood Pressure Project

This was the first evaluation of the first iteration of the Barbershop Blood Pressure Project, a barber-led intervention to support and educate men of BAME heritage in managing hypertension in London. If positive findings from the current feasibility study can be further replicated in a multisite trial, this innovative approach to health care could serve as a novel model to prevent and treat men disproportionately affected by cardiovascular and kidney disease. This evaluation provides the opportunity to address intervention limitations and strengthen the project’s future impact before widespread dissemination and implementation. Recommendations can be found in Table 2.

**Table 2.** Recommendations.

1. Alternative strategies to increase the recruitment of barbers. Previous studies have found that stylists referred by influential community leaders reinforced the importance of delivering health intervention.^25^ 2. Identify a more sustainable funding source for incentives to increase retention and motivation of barbers long-term. For example, positive outcomes and sustainability in previous barber-led BP interventions were observed when providing financial incentives per BP reading and complete client card.^14^ 3. Development of a more reliable and efficient means to record BP data. Digital recording of BP may reduce time burdens for barbers; however, study teams should also consider issues surrounding mistrust and customers’ concerns about having their data recorded. 4. Incorporate follow-up data collection to assess the effectiveness of the intervention. Follow-up surveillance of customers’ BP monitoring will require careful consideration of strategies to recruit members of the BAME community for studies. 5. Future development of the barber-led BP intervention is conducted in collaboration with all relevant stakeholders to develop an effective and culturally sensitive health intervention.

## Conclusion

The results of this study suggest that the delivery of a barber-led intervention may be a promising strategy to educate BAME men about BP and support the management of their BP. While our project begins to explore the training needs of barbers and the feasibility of implementing intervention on a small scale, future studies are needed to capture the potential benefits of barber-led interventions for BP management. Future research should consider longitudinal studies to follow up with customers, evaluate the impact of a barber-led intervention in managing BP among BAME communities and the sustainability of barber-led interventions. Given that the potential public health impact of this community-based research is high, with thousands of black-owned barbershops nationwide, a barber-led BP intervention shows promise as a novel approach to reduce health inequalities among men of BAME heritage.
